# Protocol of the IMPACT study: randomized, multicenter, phase 3 study evaluating the efficacy of immunotherapy (Atezolizumab) plus anti-VEGF therapy (Bevacizumab) in combination with transcatheter arterial chemoembolization for unresectable hepatocellular carcinoma

**DOI:** 10.1186/s12885-025-13648-5

**Published:** 2025-03-11

**Authors:** Yoshihisa Kodama, Kazuomi Ueshima, Michihisa Moriguchi, Yoshitaka Inaba, Tatsuya Yamashita, Hideki Iwamoto, Makoto Ueno, Sadahisa Ogasawara, Teiji Kuzuya, Takahiro Kodama, Yozo Sato, Toshifumi Tada, Kaoru Tsuchiya, Hideyuki Nishiofuku, Koichiro Yamakado, Miyuki Sone, Masafumi Ikeda, Tetsuo Takehara, Tetsutaro Hamano, Masatoshi Kudo

**Affiliations:** 1 Department of Radiology, Teine Keijinkai Hospital, 1-12-1-40, Maeda, Teine-Ku, Sapporo, Hokkaido 006-8555 Japan; 2 Department of Gastroenterology and Hepatology, Kindai University Faculty of Medicine, 377-2 Ohno-Higashi, Osaka-Sayama, Osaka, 589-8511 Japan; 3 Department of Molecular Gastroenterology and Hepatology, Graduate School of Medical Science, Kyoto Prefectural University of Medicine, 465, Kajii-Cho, Kawaramachi-Hirokoji, Kamigyo-Ku, Kyoto, 602-8566 Japan; 4 Department of Diagnostic and Interventional Radiology, Aichi Cancer Center Hospital, 1-1, Kanokoden, Chikusa-Ku, Nagoya, Aichi 464-8681 Japan; 5 Department of Gastroenterology, Kanazawa University Hospital, 13-1, Takara-Machi, Kanazawa, Ishikawa, 920-8641 Japan; 6 Division of Gastroenterology, Department of Medicine, Kurume University School of Medicine, 67, Asahi-Machi, Kurume, Fukuoka, 830-0011 Japan; 7 Department of Gastroenterology, Kanagawa Cancer Center, 2-3-2, Nakao, Asahi-Ku, Yokohama, Kanagawa 241-8515 Japan; 8 Department of Gastroenterology, Graduate School of Medicine, Chiba University, 1-8-1, Inohana, Chuo-Ku, Chiba, 260-8677 Japan; 9 Department of Gastroenterology and Hepatology, Fujita Health University, 1-98, Dengakugakubo, Kutsukake-Cho, Toyoake, Aichi 470-1192 Japan; 10 Department of Gastroenterology and Hepatology, Osaka University Graduate School of Medicine, 2-2, Yamadaoka, Suita, Osaka, 565-0871 Japan; 11 Department of Internal Medicine, Japanese Red Cross Society Himeji Hospital, 1-12-1, Shimoteno, Himeji, Hyogo 670-8540 Japan; 12 Department of Gastroenterology and Hepatology, Japanese Red Cross Musashino Hospital, 1-26-1, Kyonan-Cho, Musashino, Tokyo, 180-8610 Japan; 13 Department of Diagnostic and Interventional Radiology, Nara Medical University, 840, Shijo-Cho, Kashihara, Nara, 634-8522 Japan; 14 Department of Radiology, Hyogo Medical University, 1-1, Mukogawa-Cho, Nishinomiya, Hyogo 663-8501 Japan; 15 Department of Diagnostic Radiology, National Cancer Center Hospital, 5-1-1, Tsukiji, Chuo-Ku, Tokyo, 104-0045 Japan; 16 Department of Hepatobiliary and Pancreatic Oncology, National Cancer Center Hospital East, 6-5-1, Kashiwanoha, Kashiwa, Chiba, 277-8577 Japan; 17 Head office, P4 Statistics Co. Ltd., 5-11-14, Todoroki, Setagaya-Ku, Tokyo, 158-0082 Japan

**Keywords:** Atezolizumab, Bevacizumab, Conversion, Hepatocellular carcinoma, Multidisciplinary treatment, Protocol, Study design, Transcatheter arterial chemoembolization, Unresectable

## Abstract

**Background:**

Atezolizumab plus bevacizumab is recommended as a first-line treatment for unresectable hepatocellular carcinoma (uHCC). A subgroup analysis of the IMbrave150 trial showed shorter overall survival (OS) in uHCC patients with stable disease (SD) than patients with complete response (CR) or partial response (PR) after atezolizumab plus bevacizumab. Improving OS in patients with SD is an unmet medical need. Transcatheter arterial chemoembolization (TACE) may enhance treatment efficacy by controlling intrahepatic lesions and activating anti-tumor immunity. The IMPACT study aims to evaluate whether combining atezolizumab plus bevacizumab with TACE improves OS in patients with SD.

**Methods:**

IMPACT is a multicenter, phase 3 study being conducted in Japan, recruiting uHCC patients aged ≥ 18 years with Barcelona Clinic Liver Cancer stage A (single tumor ≥ 5 cm only, TACE unsuitable), stage B (TACE unsuitable) or stage C (excluding Vp3 or 4), Child–Pugh A liver function, and no prior systemic therapy. After 12 weeks of atezolizumab plus bevacizumab treatment as induction therapy, patients are being divided into two cohorts based on response: a randomized cohort for patients who achieve SD, or an atezolizumab plus bevacizumab followed by curative conversion (ABC-conversion) cohort for patients who achieve CR or PR. Patients in the randomized cohort are receiving atezolizumab plus bevacizumab and intrahepatic control TACE (Group A), or continuing atezolizumab plus bevacizumab (Group B). Patients in the ABC-conversion cohort are receiving atezolizumab plus bevacizumab. All cohorts can be considered for curative conversion therapies for residual tumors if these therapies are considered curative, in the patient's best interests, and deemed necessary by the investigator. The primary endpoint is OS for the randomized cohort and conversion rate for the ABC-conversion cohort. Secondary endpoints in both cohorts include progression-free survival, objective response rate, duration of response, time to CR, and safety. The study is expected to last 6.5 years from June 2023.

**Discussion:**

IMPACT is evaluating the efficacy of combination therapy with atezolizumab plus bevacizumab and TACE, as well as exploring the efficacy of curative conversion therapy. The results should contribute to establishing a response-guided treatment strategy for uHCC by determining optimal treatment according to the therapeutic effect of atezolizumab plus bevacizumab.

**Trial registration:**

Japan Registry of Clinical Trials (jRCT), identifier: jRCTs051230037. Registered 13 June 2023.

**Protocol version:**

8 May 2024; version 1.4.

**Supplementary Information:**

The online version contains supplementary material available at 10.1186/s12885-025-13648-5.

## Background

Primary liver cancer is the sixth most diagnosed cancer and the third most common cause of cancer deaths globally, and is associated with considerable patient and global burden [1, 2]. Geographical disparity in the impact of liver cancer is reflected in the fact that more than 50% of global cases and deaths occur in Eastern Asia [2].

Hepatocellular carcinoma (HCC), the most common histological subtype of liver cancer, was found to contribute to 80% of liver cancer cases in a 2018 population-based cancer registry survey from 95 countries [3]. Treatment recommendations for HCC are based on the Barcelona Clinic Liver Cancer (BCLC) staging system, which categorizes HCC into one of five stages, depending on the number and size of tumors: very early stage (BCLC-0), early stage (BCLC-A), intermediate stage (BCLC-B), advanced stage (BCLC-C), and terminal stage (BCLC-D) [4].

Systemic treatment is recommended for patients with BCLC-B stage HCC who have diffuse, infiltrative, and extensive bilobar liver involvement, and for patients with BCLC-C stage disease [4]. Atezolizumab (an immunotherapy that inhibits programmed death ligand 1 [PD-L1]) plus bevacizumab (a monoclonal anti-angiogenic antibody targeting vascular endothelial growth factor [VEGF]) is recommended as a first-line treatment for patients with unresectable HCC (uHCC) in the Japanese guidelines for liver cancer treatment from the Japan Society of Hepatology [5, 6], as well as the guidelines of the BCLC group [4] and the American Association for the Study of Liver Diseases [7]. These recommendations are based on the results of the IMbrave150 trial of systemic treatment-naïve patients with uHCC, in whom the combination of atezolizumab plus bevacizumab increased overall survival (OS) and progression-free survival (PFS) compared with sorafenib [8, 9]. A subgroup analysis of response data from the IMbrave150 trial found that patients with stable disease (SD) after treatment, who accounted for approximately 40% of the trial population, had a statistically significantly shorter OS than patients who achieved complete response (CR) or partial response (PR) (SD, hazard ratio [HR] 0.42; CR + PR, HR 0.11; *P* < 0.0001) [10]. Therefore, prolonging OS in patients who reach SD after initial treatment with atezolizumab plus bevacizumab is an unmet medical need in the management of uHCC. Furthermore, it would be useful to establish appropriate treatment strategies for responders and non-responders to atezolizumab plus bevacizumab.

The causes of death in HCC patients with extrahepatic metastases are liver failure or intrahepatic tumors in 72–89% of cases [11–13]. Intrahepatic tumor status and hepatic functional reserve represent major predictors of survival in patients with HCC and extrahepatic metastases [12, 13]. Transcatheter arterial chemoembolization (TACE) involves the administration of therapeutics to the liver via a catheter inserted into the hepatic artery; this method reduces or totally blocks blood supply to tumors and allows direct delivery of agents to tumors [14]. In the LAUNCH study of patients with advanced HCC, the addition of TACE to systematic treatment with lenvatinib to control intrahepatic lesions resulted in improved OS versus treatment with lenvatinib alone [15]. TACE may also activate anti-tumor immunity [16]. Thus, TACE can potentially control intrahepatic lesions and might synergistically enhance the efficacy of atezolizumab plus bevacizumab.

A multicenter proof-of-concept study found that atezolizumab plus bevacizumab combined with curative conversion therapy (i.e., resection, ablation, or super-selective TACE with curative intent following the use of atezolizumab plus bevacizumab) in TACE-unsuitable, intermediate-stage HCC patients led to the achievement of clinical CR in 35% of patients [17]. Further, 23% of patients achieved drug-free status with no recurrence, and patients with clinical CR by curative conversion therapy had prolonged survival [17]. Prospectively exploring the usefulness of curative conversion therapy in cases of response to atezolizumab plus bevacizumab will provide data that may contribute to prolonging OS and improving quality of life in patients with uHCC.

IMPACT, a phase 3 study of patients with uHCC, is aiming to investigate (i) whether combining atezolizumab plus bevacizumab with TACE can improve outcomes in patients who achieved SD after receiving atezolizumab plus bevacizumab; and (ii) the proportion of patients with CR or PR after receiving atezolizumab plus bevacizumab who subsequently achieve disease-free status (modified Response Evaluation Criteria in Solid Tumors [mRECIST] CR) with the addition of curative conversion therapy, and the prognosis of these patients.

Herein, we describe the design and rationale of the IMPACT study.

## Methods

This study protocol is reported as per the SPIRIT reporting guidelines [18].

### Objectives

The study has two objectives. Firstly, to evaluate whether the addition of intrahepatic control TACE to atezolizumab plus bevacizumab in patients who achieve SD (RECIST version 1.1 [RECIST v1.1]) after atezolizumab plus bevacizumab induction therapy will extend the duration of treatment of atezolizumab plus bevacizumab and will contribute to prolonging OS in these patients. Secondly, to exploratively investigate the prognosis and proportion of patients who achieve disease-free status (mRECIST CR) after the addition of curative conversion therapies following achievement of CR or PR with atezolizumab plus bevacizumab induction therapy.

### Patients

The study population consists of individuals aged 18 years or older with uHCC who have no history of systemic therapy for HCC, Child–Pugh class A liver function, and intrahepatic target lesions according to RECIST v1.1. Key inclusion and exclusion criteria are provided in Table 1.

**Table 1:** Key inclusion and exclusion criteria for the IMPACT study

**Inclusion criteria**
• Typical HCC based on histological/cytological examination or imaging
• Unresectable HCC, defined as: ◦ BCLC-A with a single tumor of diameter ≥ 5 cm deemed unsuitable for TACE by the investigator ◦ BCLC-B considered unsuitable for TACE by the investigator ◦ BCLC-C
• Aged 18 years or older
• At least one intrahepatic target lesion according to RECIST v1.1 criteria
• At least one intrahepatic lesion amenable to TACE that exhibits tumor staining
• No history of systemic therapy for HCC, defined as: ◦ If a history of postoperative adjuvant therapy, DFS of ≥ 6 months after the last administration date has been confirmed ◦ If a multikinase inhibitor was used concomitantly before and after TACE, DFS of ≥ 6 months from the last administration date has been confirmed
• Child–Pugh class A liver function within 14 days before enrollment
**Exclusion criteria**
• Eligible for curative conversion therapy (surgical resection, RFA) or TACE with curative intent
• Vp3 or Vp4 (right, left, or main portal vein invasion)
• Total HCC tumor volume occupies ≥ 50% of liver volume
• TACE difficult to perform or associated with high risk of complications, including patients who meet the following criteria: ◦ Severe hepatic arterial stenosis or occlusion detected by contrast CT, contrast MRI, or angiography ◦ High-grade arterio-portal or arterio-venous shunt ◦ Moderate-to-severe intrahepatic bile duct dilatation ◦ History of duodenal papillotomy or choledochojejunostomy ◦ Undergoing biliary stenting
• Current or recent (within 10 days before enrollment) use of full-dose oral or parenteral antithrombotics or thrombolytic agents for therapeutic purpose (excluding prophylaxis)
• Systemic administration of immunostimulatory agents within 180 days or immunosuppressive agents within 14 days of enrollment
• History of severe allergic or anaphylactic reactions to chimeric antibodies, humanized antibodies, or fusion proteins
• Known hypersensitivity to products derived from Chinese hamster ovary cells or to any component of atezolizumab or bevacizumab formulations

*BCLA-A* Barcelona Clinical Liver Cancer stage A (early stage), *BCLA-B* Barcelona Clinical Liver Cancer stage B (intermediate stage), *BCLA-C* Barcelona Clinic Liver Cancer stage C (advanced stage), *CT* Computed tomography, *DFS* Disease-free survival, *HCC* Hepatocellular carcinoma, *mRECIST* modified Response Evaluation Criteria in Solid Tumors, *MRI* Magnetic resonance imaging, *RECIST* Response Evaluation Criteria in Solid Tumors, *RFA* Radiofrequency ablation, *TACE* Transcatheter arterial chemoembolization, *Vp3* presence of a tumor thrombus in the first-order branches of the portal vein, *Vp4* presence of a tumor thrombus in the main trunk of the portal vein or a portal vein branch contralateral to the primarily involved lobe (or both)

Patients who meet the inclusion criteria and who provide written informed consent to participate in the study are being recruited and enrolled using an electronic data capture (EDC) system.

### Study design and setting

**Fig. 1:**
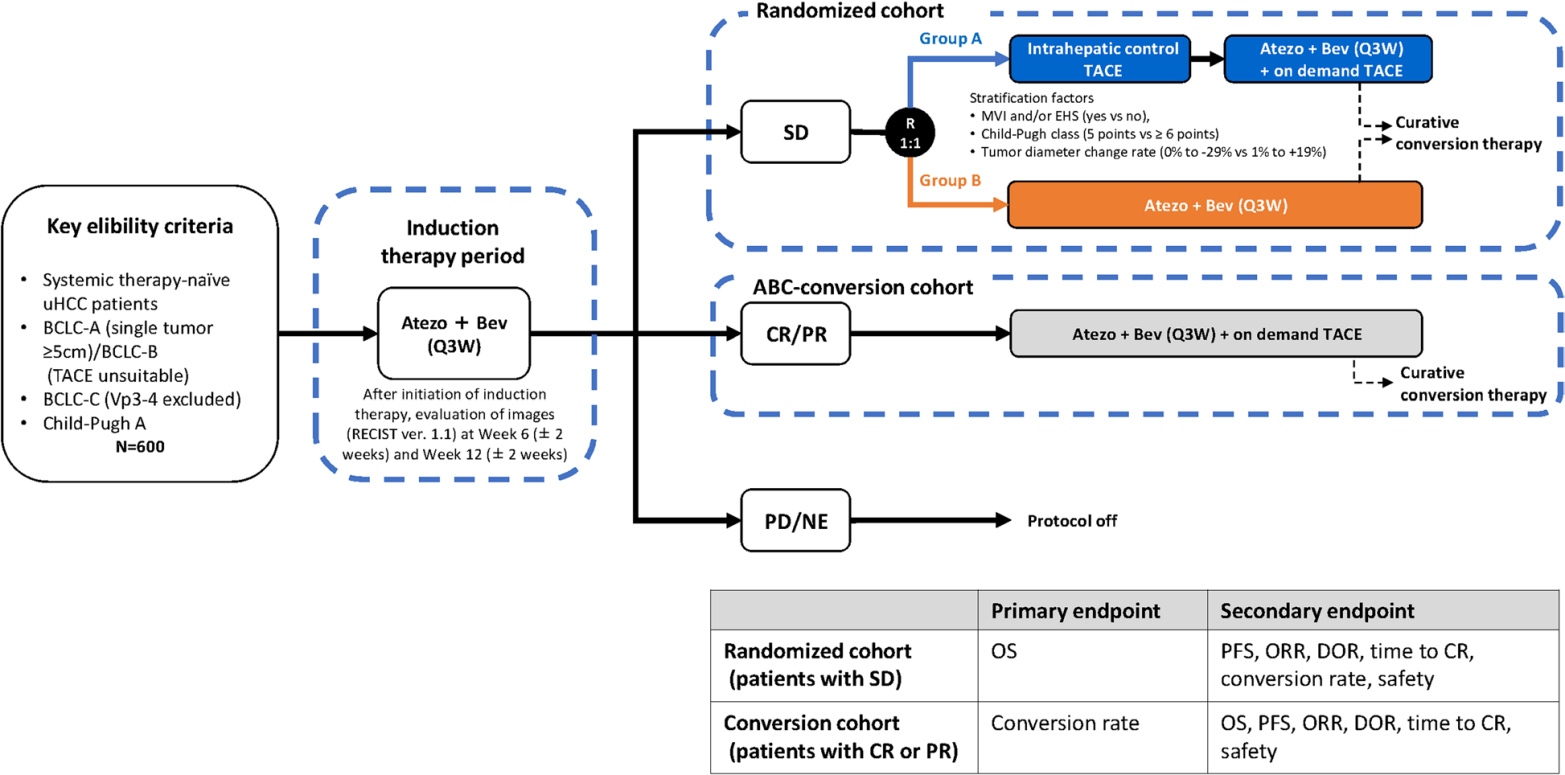
Study schema of the IMPACT study. PFS is defined as the shortest time to first disease progression (determined using RECIST v1.1 or mRECIST) or death from any cause from the date of randomization. ORR is defined as the proportion of patients in whom the best overall response is either CR or PR from the date of initiation of induction therapy to the date of discontinuation of protocol treatment or the date of first observed progression or death, whichever occurs first. DOR is defined as the time from the date of the first confirmed response (the date of first documented CR or PR status) to the date of the first confirmed disease progression or death, whichever occurs first, after transition to the randomized cohort. Time to CR is defined as the time from randomization to the first occurrence of CR (determined using RECIST v1.1). ABC-conversion, atezolizumab plus bevacizumab followed by curative conversion; Atezo, atezolizumab; Bev, bevacizumab; BCLC-A, Barcelona Clinic Liver Cancer stage A (early stage); BCLC-B, Barcelona Clinic Liver Cancer stage B (intermediate stage); BCLC-C, Barcelona Clinic Liver Cancer (advanced stage); CR, complete response; DOR, duration of response; EHS, extrahepatic spread; MVI, macroscopic vascular invasion; NE, not evaluable; ORR, objective response rate; OS, overall survival; PD, progressive disease; PFS, progression-free survival; PR, partial response; Q3W, every 3 weeks; R, randomization; (m)RECIST v1.1, (modified) Response Evaluation Criteria in Solid Tumors version 1.1; SD, stable disease; TACE, transcatheter arterial chemoembolization; uHCC, unresectable hepatocellular carcinoma; Vp3-4, right, left, or main portal vein invasion

IMPACT is an interventional, randomized, phase 3, multicenter study being conducted in Japan. The investigators will assign enrolled patients to one of two cohorts according to their response to initial treatment with atezolizumab plus bevacizumab during a 12-week induction therapy period: a randomized cohort, or an atezolizumab plus bevacizumab followed by curative conversion (ABC-conversion) cohort (Fig. 1). The criteria for transition to either the randomized cohort or the ABC-conversion cohort is based on the response to induction therapy assessed by the investigator using imaging (chest through pelvis computed tomography [CT], abdominal dynamic contrast CT, and abdominal dynamic contrast magnetic resonance imaging [MRI]) at 12 weeks (± 2 weeks) compared with images taken before induction therapy using RECIST v1.1. Patients with SD are being transitioned to the randomized cohort, while those with CR/PR are being transitioned to the ABC-conversion cohort. Patients with progressive disease (PD) or who are not evaluable with imaging after 12 weeks of induction therapy are taken off the protocol and excluded from further inclusion in the study. An early transition to the randomized cohort based on imaging assessment at 6 (+ 2) weeks is possible and has been designed for patients with SD who meet the following criteria: target lesion size tends to increase, or elevated alpha-fetoprotein (AFP) levels, or AFP-L3 fraction (%) becomes positive.

Randomization is in a 1:1 ratio using an EDC system program, stratified by macroscopic vascular invasion and/or extrahepatic spread (yes vs no), Child–Pugh score (5 points vs ≥ 6 points), and percent change in the sum of longest diameter (SLD) of target lesions from baseline (0% to − 29% vs 1% to + 19%). Study participants and investigators are not blinded to allocated treatment.

AFP level was not included as a stratification factor in this study. This decision was based on the trial design, which involved randomization according to treatment response after initiation of 2–4 cycles of induction therapy. Although baseline AFP level is a well-established prognostic factor in HCC, the prognostic value of post-treatment AFP levels has not been established for uHCC.

### Treatments

#### Induction period

During the induction period, patients receive atezolizumab 1200 mg plus bevacizumab 15 mg/kg by intravenous infusion on a 3-week cycle (drugs administered on Day 1 of the 21-day cycle) for 12 weeks.

#### Randomized cohort

Patients in the randomized cohort are receiving atezolizumab plus bevacizumab with intrahepatic control TACE (Group A), or continuing to receive treatment with atezolizumab plus bevacizumab (Group B).

**Fig. 2:**
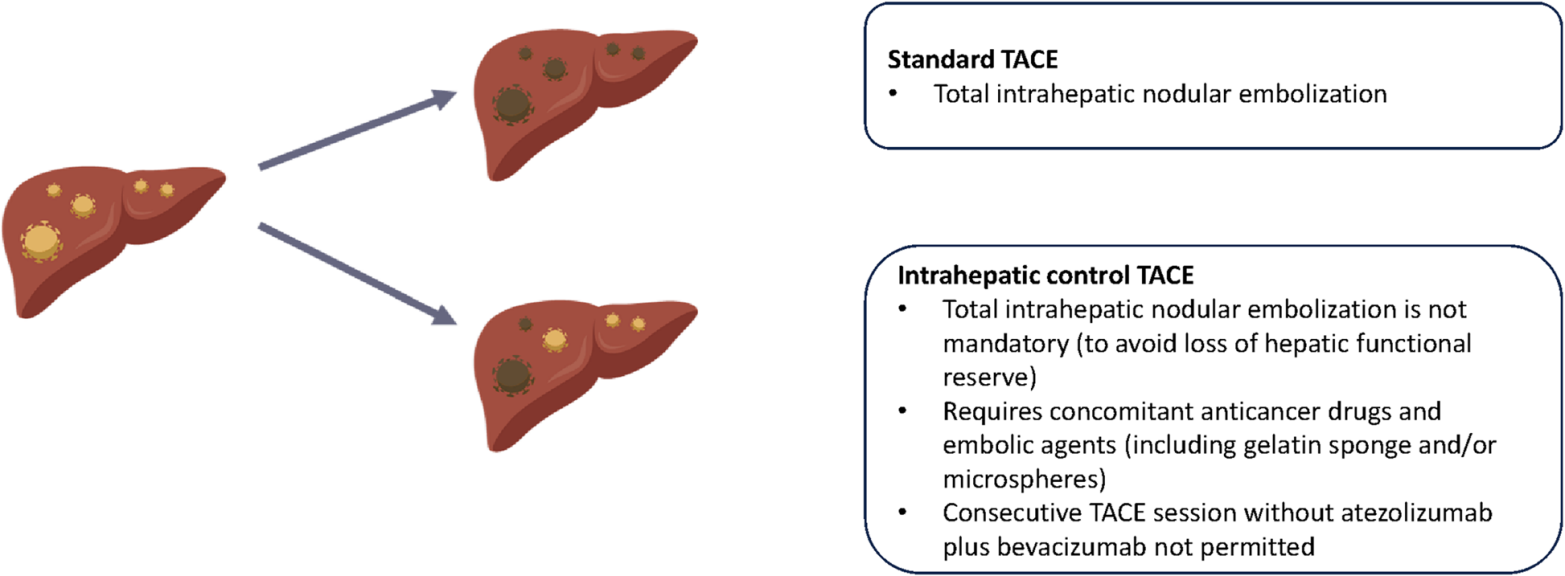
Intrahepatic control transcatheter arterial chemoembolization (TACE), versus standard TACE. The purpose of intrahepatic control TACE is to reduce tumor burden, while preserving hepatic functional reserve. Further, the release of neoantigens may enhance the efficacy of atezolizumab plus bevacizumab

Intrahepatic control TACE, with the purpose of reducing intrahepatic tumor burden while minimizing effects on hepatic functional reserve (Fig. 2), is being performed in Group A of the randomized cohort. It involves the embolization (using gelatin sponge or microspheres) of 1–3 prognostically relevant tumor nodules and administration of anti-cancer drugs (epirubicin hydrochloride, cisplatin, and miriplatin hydrate). After administration of atezolizumab plus bevacizumab (Day 1), intrahepatic control TACE is performed during Days 15–28. After TACE, a period of ≥ 7 days is required before restarting atezolizumab plus bevacizumab, and two consecutive sessions of TACE without atezolizumab plus bevacizumab are not permitted.

On-demand TACE is allowed if the remaining intrahepatic lesions or tumor markers (AFP, des-gamma-carboxy-prothrombin, or AFP-L3) increase or if the investigators consider it likely to be clinically effective for patients in Group A.

Curative conversion therapy for residual tumor is acceptable for patients in Group A and Group B if it is considered that the tumor has decreased in size, the therapy will be curative and it will be in the patient’s best interests and is deemed clinically necessary by the investigator. Atezolizumab plus bevacizumab is being continued until the following three conditions (‘drug-off criteria’) have continued for ≥ 24 weeks (or ≥ 12 weeks in the case of surgical resection): (i) achievement of CR per mRECIST on CT or MRI; (ii) normalization of tumor markers (AFP/AFP-L3/des-gamma-carboxy prothrombin); and (iii) complete disappearance of intramodular arterial flow as evaluated by contrast-enhanced ultrasonography. If the drug-off criteria are met, the decision to discontinue atezolizumab plus bevacizumab is at the discretion of the investigator and discontinuation is not mandatory.

#### ABC-conversion cohort

Patients in the ABC-conversion cohort are continuing to receive the atezolizumab plus bevacizumab treatment. The ABC-conversion cohort may also receive curative conversion therapy for residual disease in addition to atezolizumab plus bevacizumab. Curative conversion therapy includes surgical resection, radiofrequency ablation (RFA)/microwave ablation, TACE with curative intent, and X-ray/particle radiotherapy (Table 2). Criteria for follow-up, consideration for on-demand TACE, and imaging to determine response to treatment are consistent with those for Group A of the randomized cohort.

**Table 2:** Curative conversion therapy in the ABC-conversion cohort of the IMPACT study

**Type of curative conversion therapy**	**Timing of curative conversion therapy**	**Resumption of atezolizumab plus bevacizumab**^c^
Surgical resection	Bevacizumab withdrawn for one cycle immediately before surgical resection Surgery should be performed ≥ 3 weeks from the last administration date of atezolizumab (≥ 6 weeks from last administration date of bevacizumab)	After 28 days following surgical resection, atezolizumab plus bevacizumab should be resumed after confirming that the surgical wound is normal (must be within 12 weeks of the last dose of atezolizumab plus bevacizumab)
RFA/MWA TACE with curative intent^a^	≥ 2 weeks after the last dose of atezolizumab plus bevacizumab	On or after Day 7, atezolizumab plus bevacizumab should be resumed after confirming the puncture site is normal (must be within 12 weeks of the last dose of atezolizumab plus bevacizumab)
X-ray/particle radiotherapy^b^	On careful judgment	On careful judgment

*ABC-conversion* atezolizumab plus bevacizumab followed by curative conversion, *MWA* Microwave ablation, *RFA* Radiofrequency ablation, *TACE* Transcatheter arterial chemoembolization

^a^Performed only when all nodules can be embolized with a single TACE session

^b^X-ray/particle radiotherapy should be carefully evaluated based on the irradiation site

^c^Treatment is resumed after curative conversion therapy until the participant meets the ‘drug-off’ criteria

#### Concomitant therapies

Concomitant therapies that are permitted include those used for the treatment of adverse events (AEs), at the discretion of the investigator; symptomatic treatment that had been initiated prior to the start of the study; and combination therapy to relieve symptoms such as worsening of bone metastasis pain. Therapies that are prohibited include combination therapy for the treatment of cancer that is not included in the protocol therapy; drugs and therapies contraindicated in combination with anticancer drugs used in the protocol therapy; and investigational products and unapproved drugs.

### Assessments

Imaging to determine response to treatment (using RECIST v1.1 and mRECIST) are being performed every 6 (± 2) weeks until Week 24, and every 9 (± 2) weeks thereafter. In this study, PD is confirmed by consecutive imaging evaluation performed 6 weeks (± 2 weeks) after the initial confirmation of PD. However, if a new lesion is identified, it will be assessed as PD.

The severity of AEs is being assessed according to the Common Terminology Criteria for Adverse Events from the Japanese Cooperative Oncology Group (CTCAE v 5.0-JCOG) classification.

### Outcomes

The primary outcome is composed of two primary efficacy endpoints: OS in the randomized cohort, which is defined as the period from the date of transition to the randomized cohort to the date of death from any cause; and the rate of conversion in the ABC-conversion cohort, which is defined as the percentage of patients who achieve disease-free status (mRECIST CR assessed by CT/MRI) with the addition of curative conversion therapy (surgical resection, RFA, TACE with curative intent, etc.) after atezolizumab plus bevacizumab among all patients transferred to the ABC-conversion cohort.

The secondary endpoints in both cohorts include PFS, objective response rate (ORR), duration of response (DOR), and time to CR. Safety is also a secondary endpoint in both cohorts. Conversion rate is a secondary endpoint in the randomized cohort, and OS is a secondary endpoint in the ABC-conversion cohort (Fig. 1).

The definitions of the secondary endpoints are provided in the Supplementary Methods section of the Supplementary Material.

### Sample size

The planned sample sizes are 600 patients in the induction phase and 315 patients in the randomization cohort at 99 departments in 98 facilities. These sample sizes were set based on the results of the IMbrave150 trial [8]. The planned number of enrolled patients in the ABC-conversion cohort is not set; however, the cohort is expected to include approximately 110 patients.

The study is currently recruiting and, as of June 2024, 188 patients have been enrolled. It is anticipated that the enrollment period will be 2.5 years from the date of registration of the study in the Japan Registry of Clinical Trials (jRCTs051230037), the follow-up period will be 2.5 years from the enrollment date of the last patient, and the total study duration will be 6.5 years from jRCT registration.

Further details of the rationale for the sample sizes are provided in the Supplementary Methods section of the Supplementary Material.

### Statistical methods

The final primary analysis of OS will be performed for all transitioned patients (intention-to-treat) in the randomized cohorts. Patients alive at the time of data analysis cut-off will be censored at the last known date of survival. A stratified log-rank test will be used to compare OS in Group A and Group B. The HR and its 95% confidence interval (CI) will be estimated using a stratified Cox model. The Kaplan–Meier method will be used to estimate OS and its 95% CI.

An interim analysis is planned after 124 OS events have been observed in both treatment groups of the randomized cohort (Group A and Group B) combined. The difference in OS for the randomization cohort will be compared using a stratified log-rank test, with the stratification factors used for randomization. The HR and its 95% CI will be estimated using a stratified Cox model.

For the primary analysis, a two-sided overall significance level will be set to 5%. The significance level to the interim analysis and the final analysis will be allocated using the Lan-DeMets method with O’Brien-Fleming type alpha spending function. The interim analysis will include analysis of the primary outcome (OS) in the randomized cohort, as well as of the secondary endpoints, and will be performed by a statistical analyst who is independent of the statistical analyst who will perform the final analysis; based on the results, an Independent Efficacy and Safety Evaluation Committee will determine whether the study should continue or be discontinued.

In the ABC-conversion cohort, the primary endpoint is conversion rate. The rate and its 95% CI will be estimated.

Methods of statistical analysis used for the secondary endpoints are provided in the Supplementary Methods section of the Supplementary Material.

### Data collection and monitoring

Study investigators are maintaining records of treatment administration and adherence to treatment via case report forms (CRFs), preferably in an electronic form, although paper CRFs are used if needed. CRF data is entered into the EDC system (Viedoc™ Ver 4.77), and the data center is responsible for data management (including quality check, back-up and storage), with data entry into the EDC the responsibility of each institution. Any corrections to the CRFs will be followed via an audit trail in the EDC. The data entered into the CRFs is being audited regularly via remote assessment by a separate auditing/monitoring organization (funded by the study sponsor) to ensure that treatment is administered according to the protocol. Auditing of the study sites is also being performed by this organization; the auditors conduct site visits and prepare a report for the principal investigator and study office, and the investigator at each site.

Study data will be retained for five years after the discontinuation or completion of the study or three years after the final report of the study results, whichever is later. At the end of the retention period, documents will be appropriately disposed of, in accordance with the rules of the participating institution.

### Ethics and dissemination

The study protocol was reviewed and approved by the Nara Medical University Certified Review Board (ethics approval number nara0058). Any amendments or revisions to the protocol must receive approval from a certified review board; the changes will then be registered in the jRCT and the Minister of Health, Labour and Welfare notified, and reconsent from enrolled participants may be required.

The study is being conducted in accordance with the principles of the Declaration of Helsinki, and the Ministry of Health, Labour and Welfare’s Clinical Trials Act and Ordinance for Enforcement of the Clinical Trials Act. All potential participants receive written and oral information about the study prior to enrolment; written informed consent is obtained from all participants by the investigators.

If an AE occurs, treatment of the AE will be provided in accordance with usual medical care. In the event that a participant suffers a health injury as a result of participation in the IMPACT study, compensation will be provided, depending on the nature of the injury and determination of causality by the investigator.

Personal information (names) of the enrolled study participants will not be provided by the institution to the support office or data center; participants are identified and referred to using a registration number, month of birth, and an identification code.

Once the main results of the trial are published, the final study dataset (anonymized) will be available to researchers upon reasonable request.

Independent of the efficacy and safety observed, the results of the primary analysis and secondary analyses will be disseminated. An overview, progress report, and main results will be published in the jRCT. The results of the study will be summarized in a paper that will be submitted to a peer-reviewed journal; the authors will be required to meet all four authorship requirements of the International Committee of Medical Journal Editors. Data from the study may be used for secondary purposes (e.g., for use in a meta-analysis). Further, the results of the study will be communicated to the participants via a written report, and, upon request, participants may receive a report on their personal result written in plain language.

After the publication of the main analysis paper, a document for patients and the general public (i.e., a lay summary) will be prepared; public access to the full protocol, participant-level dataset and statistical code is not planned.

## Discussion

The IMPACT study, which is currently recruiting (as of June 2024), is investigating approaches to improving outcomes in patients with uHCC who have been treated with atezolizumab plus bevacizumab. While atezolizumab plus bevacizumab is a current first-line treatment for uHCC, objective response (CR + PR) was seen in only 30% of patients treated with this combination and the survival rate at 18 months was approximately 50% in the pivotal IMbrave150 trial [9]. Thus, there is a need for more effective treatment in the substantial proportion of patients who do not experience a full clinical response to atezolizumab plus bevacizumab. It is hoped that the results of the IMPACT study will provide data to guide treatment in these patients.

One of the unique features of the IMPACT study is the use of intrahepatic control TACE. TACE is recommended for the management of patients with intermediate stage HCC [4, 5, 7], but, when using this therapy, the control of intrahepatic lesions must be balanced with the maintenance of hepatic functional reserve. Standard TACE aims for total intrahepatic nodular embolization, which carries the risk of a decrease in hepatic functional reserve. In contrast, intrahepatic control TACE takes hepatic functional reserve into account, by mainly focusing on embolization of prognosis-defining lesions, to minimize effects on hepatic functional reserve. It should be noted that in the IMPACT study TACE is being administered following atezolizumab plus bevacizumab induction therapy, and prior to resumption of atezolizumab plus bevacizumab. This sequential approach (rather than combined administration of TACE with atezolizumab plus bevacizumab initially) has been chosen because some lesions may respond well during the atezolizumab plus bevacizumab induction therapy period. Therefore, use of TACE after induction therapy allows the selective embolization of lesions that have not responded to initial treatment with atezolizumab plus bevacizumab induction and that are likely to determine prognosis.

Regarding the rationale for other aspects of the IMPACT study design, although the timing of randomization is primarily set at 12 weeks (based on the median time to response to atezolizumab plus bevacizumab of 2.8 months observed in the IMbrave150 study [19]), patients can also be randomized at 6 weeks post-induction with atezolizumab plus bevacizumab. This early randomization is reserved for patients who have achieved SD but are considered to have a particularly poor prognosis, i.e., those showing a trend for tumor enlargement of + 1 to + 19% at the 6-week point (it should be noted that the RECIST v1.1 category of SD includes patients with percent tumor diameter change ranging from − 29% to + 19% versus baseline, encompassing both patients with tumor shrinkage and those with tumor enlargement). Also, the stratification factors for the SD randomized cohort include percent change in SLD of target lesions from baseline (0% to − 29% vs. + 1% to + 19%). This is included as a factor because the results from the IMbrave150 trial suggest that change in SLD of target lesions from baseline has a significant impact on OS [20], the primary endpoint in the SD cohort in the IMPACT study. Further, analysis of data from the IMbrave150 trial suggests that patients with a tumor shrinkage trend (0% to − 29%) at 12 weeks may include those who will later achieve PR. Since patients with CR/PR have a better prognosis than patients with SD, percent change in tumor diameter has been included as a stratification factor to prevent imbalance in patients with a tumor shrinkage trend at 12 weeks.

The IMPACT study is assessing the percentage of patients who achieve conversion to cancer-free status after receiving atezolizumab plus bevacizumab therapy, and their prognosis. If the results for this endpoint are promising, they may indicate that curative conversion therapy should be actively recommended to patients with uHCC, and serve as important reference data for future studies.

A number of limitations of the IMPACT study should be considered. Firstly, due to ethical considerations, in the randomized cohort, Group B can undergo TACE only if TACE with curative intent is feasible. This may potentially dilute the treatment outcomes of Group A, which aims to extend patient prognosis by combining atezolizumab plus bevacizumab with TACE. Secondly, this study uses intrahepatic control TACE, a new concept in TACE, and its use may not be consistent across participating institutions, as investigators at each institution can decide which nodule(s) to embolized to ensure minimal impact on hepatic functional reserve. Thirdly, the ABC-conversion cohort is employing a single-arm trial design without setting hypotheses, meaning it is not possible to verify whether adding curative conversion therapy truly prolongs patient prognosis. Finally, changes in biomarkers are not being investigated and, thus, it will not be possible to determine whether the combination of drug therapy and TACE has a synergistic effect on activation of anti-tumor immunity through the release of tumor antigens.

Despite these limitations, we believe that the knowledge gained from the IMPACT study should assist clinicians in managing uHCC, and improve outcomes for patients with this common form of liver cancer.

## Supplementary Information

Supplementary Material 1. Supplementary Methods (sample size calculations; statistical analysis).

## Data Availability

No datasets were generated or analysed during the current study.

## Abbreviations

ABC-conversion: Atezolizumab plus bevacizumab followed by curative conversion
AE: Adverse events
AFP: Alpha-fetoprotein
BCLC: Barcelona Clinic Liver Cancer
BCLC-0: Very early stage liver cancer
BCLC-A: Early stage liver cancer
BCLC-B: Intermediate stage liver cancer
BCLC-C: Advanced stage liver cancer
BCLC-D: Terminal stage liver cancer
CI: Confidence interval
CR: Complete response
CRF: Case report form
CT: Computed tomography
CTCAE v 5.0-JCOG: Common Terminology Criteria for Adverse Events from the Japanese Cooperative Oncology Group
DFS: Disease-free survival
DOR: Duration of response
EDC: Electronic data capture
HCC: Hepatocellular carcinoma
HR: Hazard ratio
jRCT: Japan Registry of Clinical Trials
MRI: Magnetic resonance imaging
ORR: Objective response rate
OS: Overall survival
PD: Progressive disease
PD-L1: Programmed death ligand 1
PFS: Progression-free survival
PR: Partial response
(m)RECIST: (Modified) Response Evaluation Criteria in Solid Tumors
RFA: Radiofrequency ablation
SD: Stable disease
SLD: Sum of longest diameter
TACE: Transcatheter arterial chemoembolization
uHCC: Unresectable hepatocellular carcinoma
VEGF: Vascular endothelial growth factor
Vp3: Presence of a tumor thrombus in the first-order branches of the portal vein
Vp4: Presence of a tumor thrombus in the main trunk of the portal vein or a portal vein branch contralateral to the primarily involved lobe (or both)
