# Supplementary material for: Protocol of the IMPACT study: randomized, multicenter, phase 3 study evaluating the efficacy of immunotherapy (Atezolizumab) plus anti-VEGF therapy (Bevacizumab) in combination with transcatheter arterial chemoembolization for unresectable hepatocellular carcinoma
Source: BMC Cancer. 2025 Mar 11;25:434. doi: 10.1186/s12885-025-13648-5 (PMC11895279; doi:10.1186/s12885-025-13648-5)
Supplement: Supplementary file 1 — Supplementary Material 1. Supplementary Methods (sample size calculations; statistical analysis). [file 12885_2025_13648_MOESM1_ESM.docx]

**Protocol of the IMPACT study: Randomized, multicenter, phase 3 study evaluating the efficacy of immunotherapy (Atezolizumab) plus anti-VEGF therapy (Bevacizumab) in combination with transcatheter arterial chemoembolization for unresectable hepatocellular carcinoma**

**Authors:** Yoshihisa Kodama^1^, Kazuomi Ueshima^2^, Michihisa Moriguchi^3^, Yoshitaka Inaba^4^, Tatsuya Yamashita^5^, Hideki Iwamoto^6^, Makoto Ueno^7^, Sadahisa Ogasawara^8^, Teiji Kuzuya^9^, Takahiro Kodama^10^, Yozo Sato^4^, Toshifumi Tada^11^, Kaoru Tsuchiya^12^, Hideyuki Nishiofuku^13^, Koichiro Yamakado^14^, Miyuki Sone^15^, Masafumi Ikeda^16^, Tetsuo Takehara^10^, Tetsutaro Hamano^17^, and Masatoshi Kudo^2^

**Affiliations:** ^1^Department of Radiology, Teine Keijinkai Hospital, 1-12-1-40, Maeda, Teine-ku, Sapporo, Hokkaido, 006-8555, Japan; ^2^Department of Gastroenterology and Hepatology, Kindai University Faculty of Medicine, 377-2 Ohno-Higashi, Osaka-Sayama, Osaka 589-8511, Japan; ^3^Department of Molecular Gastroenterology and Hepatology, Graduate School of Medical Science, Kyoto Prefectural University of Medicine, 465, Kajii-cho, Kawaramachi-Hirokoji, Kamigyo-ku, Kyoto, 602-8566, Japan; ^4^Department of Diagnostic and Interventional Radiology, Aichi Cancer Center Hospital, 1-1, Kanokoden, Chikusa-ku, Nagoya, Aichi, 464-8681, Japan; ^5^Department of Gastroenterology, Kanazawa University Hospital, 13-1, Takara-machi, Kanazawa Ishikawa, 920-8641, Japan; ^6^Division of Gastroenterology, Department of Medicine, Kurume University School of Medicine, 67, Asahi-machi, Kurume, Fukuoka, 830-0011, Japan; ^7^Department of Gastroenterology, Kanagawa Cancer Center , 2-3-2, Nakao, Asahi-ku, Yokohama, Kanagawa, 241-8515, Japan; ^8^Department of Gastroenterology, Graduate School of Medicine, Chiba University, 1-8-1, Inohana, Chuo-ku, Chiba, Chiba, 260-8677, Japan; ^9^Department of Gastroenterology and Hepatology, Fujita Health University, 1-98, Dengakugakubo, Kutsukake-cho, Toyoake, Aichi, 470-1192, Japan; ^10^Department of Gastroenterology and Hepatology, Osaka University Graduate School of Medicine, 2-2, Yamadaoka, Suita, Osaka, 565-0871, Japan; ^11^Department of Internal Medicine, Japanese Red Cross Society Himeji Hospital, 1-12-1, Shimoteno, Himeji, Hyogo, 670-8540, Japan; ^12^Department of Gastroenterology and Hepatology, Japanese Red Cross Musashino Hospital, 1-26-1, Kyonan-cho, Musashino-shi, Tokyo, 180-8610, Japan; ^13^Department of Diagnostic and Interventional Radiology, Nara Medical University, 840, Shijo-Cho, Kashihara, Nara, 634-8522, Japan; ^14^Department of Radiology, Hyogo Medical University, 1-1, Mukogawa-cho, Nishinomiya, Hyogo, 663-8501, Japan; ^15^Department of Diagnostic Radiology, National Cancer Center Hospital, 5-1-1, Tsukiji, Chuo-ku, Tokyo, 104-0045, Japan; ^16^Department of Hepatobiliary and Pancreatic Oncology, National Cancer Center Hospital East, 6-5-1, Kashiwanoha, Kashiwa, Chiba, 277-8577, Japan; ^17^Head Office, P4 Statistics Co. Ltd., 5-11-14, Todoroki, Setagaya-ku, Tokyo, 158-0082, Japan

**Corresponding author:** M Kudo, Department of Gastroenterology and Hepatology, Kindai University Faculty of Medicine, 377-2 Ohno-Higashi, Osaka-Sayama, Osaka 589-8511, Japan. Email: m-kudo@med.kindai.ac.jp

**Supplementary materials**

**Supplementary Methods**

***Sample size calculations***

In the IMbrave150 trial [1], the best overall response to atezolizumab plus bevacizumab was determined to be stable disease (SD) in approximately 47% of patients at a median follow-up of 8.9 months; however, to assess SD at an earlier timepoint in the IMPACT study, it was assumed that 60% of patients would achieve SD. With a two-sided significance level of 0.05, a power of 90%, an enrollment period of 2.5 years, a follow-up period of 2.5 years, an expected hazard ratio (HR) of 0.66 for overall survival (OS) in the group receiving TACE versus the non-TACE group (based on OS in the IMbrave150 trial [1]), and an interim analysis of OS planned after 124 events were observed (using an O’Brien-Fleming alpha spending function), the number of events required was estimated to be 245. The number of patients transitioning to the randomization cohort required to achieve this number of events was estimated to be 300; thus, the planned number of patients transitioning to the randomization cohort has been set at 315, assuming a 5% dropout. Thus, the planned enrollment size for the induction phase is 600 patients, allowing for a 10% dropout from enrollment to the randomization cohort transition, including discontinuation of induction therapy.

The planned number of enrolled patients in the ABC-conversion cohort is not set. However, based on complete response (CR) and partial response (PR) rates of approximately 6% and 22% (according to Response Evaluation Criteria in Solid Tumors version 1.1 [RECIST v1.1]), respectively, at a median follow-up of 8.9 months in the IMbrave150 trial [1], a CR and PR rate of 20% at an earlier timepoint in the IMPACT study has been assumed. This cohort is expected to accumulate approximately 110 patients, considering a 10% dropout.

***Statistical analysis***

*Secondary endpoints*

Secondary endpoints will be analyzed to supplement the results of the primary analysis of the study, but due to their exploratory nature, multiplicity will not be adjusted for unless specified in the statistical analysis plan in the interim analysis.

Analysis of secondary endpoints in randomized cohorts

Progression-free survival (PFS) is defined as the shortest time to first disease progression or death from any cause, from the date of randomization. Progression is determined by the investigators based on RECIST v1.1 or modified RECIST (mRECIST). The comparison of PFS between Group A and Group B, estimation of median PFS and its 95% confidence interval (CI), and calculation of HR and its 95% CI will be similar to that for the analysis of the primary outcomes in the randomized cohorts.

The objective response rate (ORR) is defined as the proportion of patients in whom the best overall response is either CR or PR from the date of initiation of induction therapy to the date of discontinuation of protocol treatment or the date of first observed disease progression or death, whichever occurs first, among all patients transitioning in the randomized cohorts who had measurable disease at enrollment. The ORR and its 95% CI will be calculated for each group for RECIST v1.1 or mRECIST as determined by the investigators. Hypothesis testing for the ORR will not be performed.

The duration of response (DOR) includes responders from the entire randomized cohort with measurable disease at enrollment. The DOR is defined as the time from the date of the first confirmed response (the date of the first documented CR or PR status) to the date of the first documented disease progression or death, whichever occurs first, after the transition to the randomized cohort. Patients without disease progression at the cut-off date and who have not died will be censored at the cut-off date or at the last tumor evaluation before that date. DOR analyses will be evaluated by the investigators according to RECIST v1.1 and mRECIST. Comparisons of DOR between Group A and Group B, estimation of median DOR and 95% CI, and calculation of HR and 95% CI will be similar to those for the analysis of the primary outcomes in the randomized cohort.

Time to CR will be determined for all transitioning patients in the randomized cohorts with measurable disease at enrollment. Time to CR is defined as the time from randomization to the first occurrence of CR (determined by the investigators based on RECIST v1.1). Patients who do not have a CR by the time of protocol discontinuation or the cut-off date will be censored at the time of tumor evaluation immediately before the discontinuation or cut-off date, whichever occurs first. Patients without a post-baseline evaluation will be censored at the date of randomization. The comparison of the two groups for time to CR, estimation of the median time to CR and 95% CI, and calculation of the HR and its 95% CI will be similar to those for the analysis of the primary outcomes in the randomized cohort.

The conversion rate is defined as the number of patients who underwent curative conversion therapy (e.g., surgical resection, radiofrequency ablation [RFA], transcatheter arterial chemoembolization [TACE] with curative intent) who achieved mRECIST CR among all patients transitioning in the randomized cohort. The percentage (and 95% CI) will be calculated using a denominator of the number of transitioning patients in the randomized cohorts and a numerator of the number of patients who underwent curative therapy.

The incidence of adverse events (AEs; proportion of patients with the worst grade of AE), AEs of special interest (proportion of patients) and AEs with suspected autoimmune involvement (irAEs; proportion of patients) will be calculated using the safety analysis set as the denominator. To evaluate the changes in Child-Pugh score and albumin-bilirubin (ALBI)/modified ALBI (mALBI) score, evaluations will be made at baseline and at the beginning of the course in the safety analysis set. The percentage of patients treated with steroid therapy for irAEs will be calculated using the number of patients with irAEs as the denominator.

Analysis of secondary endpoints in the ABC-conversion cohort

OS is defined as the period from the date of transition to the ABC-conversion cohort to the date of death from any cause. Patients still alive at the time of data addition will be censored at the last known date of survival. Analyses of OS will be performed on all patients in the ABC-conversion cohort. Kaplan-Meier methods will be used to estimate OS and its 95% CI, and, if available, median OS and its 95% CI will be calculated.

PFS is defined as the shortest time to first exacerbation or death from any cause, beginning with the ABC-conversion cohort transition date. Disease progression will be determined by the investigators based on RECIST v1.1 or mRECIST. Patients with no documented progression or death from any cause at the time of data addition will be censored at the last date of documented progression-free survival. Analysis of PFS will be performed for all ABC-conversion cohorts. Estimation of PFS, median PFS, and calculation of 95% CI will be similar to the analysis of OS in the ABC-conversion cohort.

The ORR – for all transitioning ABC-conversion cohorts with measurable disease at enrollment – is defined as the proportion of patients whose best overall response is either CR or PR from the start of induction therapy to the end of protocol therapy or the date of first observed disease progression or death, whichever occurs first. The ORR and its 95% CI will be calculated for each group for RECIST v1.1 or mRECIST as determined by the investigators. Hypothesis testing for the ORR will not be performed.

The DOR will include all patients in the ABC-conversion cohort with measurable disease at enrollment. The DOR is defined as the period from the date of first documented response (the date of first documented CR or PR status) to the date of first documented disease progression or death, whichever occurs first. Patients without disease progression or death at the cutoff date will be censored at the cutoff date or at the last tumor assessment before that date. Analysis of DOR will be evaluated by the investigators according to RECIST v1.1 and mRECIST. Estimation of DOR, median DOR and calculation of 95% CI will be similar to the analysis of OS in ABC-conversion cohort.

The time to CR will be determined for PR among all ABC-conversion patients with measurable disease at enrollment. Time to CR is defined as the time from ABC-conversion to the first occurrence of CR. CR will be determined by the investigators based on RECIST v1.1 or mRECIST. Patients who do not have a CR by the time of discontinuation of protocol therapy or the cutoff date will be censored at the time of the tumor evaluation immediately preceding the discontinuation or cutoff date, whichever occurs first. Patients who do not have a post-ABC-conversion transition evaluation will be censored at the ABC-conversion transition date. Time to CR, estimation of median time to CR, and calculation of 95% CI will be similar to the analysis of OS in the ABC-conversion cohort.

**References**

1. Finn RS, Qin S, Ikeda M, Galle PR, Ducreux M, Kim TY, et al. Atezolizumab plus bevacizumab in unresectable hepatocellular carcinoma. N Engl J Med. 2020;382(20):1894-905.
